# Evaluation of Serum Levels of IL-17 and IL-22 in Leprosy: A Case-Control Study

**DOI:** 10.7759/cureus.103563

**Published:** 2026-02-13

**Authors:** Arushi Nanda, Aneet Mahendra, Aditi Dabhra, Sanjeev Gupta

**Affiliations:** 1 Dermatology, Maharishi Markandeshwar Institute of Medical Sciences and Research (Deemed to be University), Ambala, IND

**Keywords:** interleukin-17, interleukin-22, leprosy, leprosy reaction, ridley-jopling classification

## Abstract

Background: Leprosy exhibits a wide clinical spectrum that is largely determined by host immune responses. Recent evidence suggests an important role of the Th17/Th22 axis in disease activity and lepra reactions.

Primary objective: This study aimed to compare serum interleukin (IL)-17 and IL-22 levels between patients with leprosy and age- and sex-matched healthy controls.

Secondary objectives: This study also aimed (i) to evaluate the variation of serum IL-17 and IL-22 levels across the Ridley-Jopling spectrum of leprosy; (ii) to assess their association with lepra reactional states; and (iii) to explore the relationship of these cytokines with slit-skin smear status and disease duration.

Methods: This cross-sectional case-control study included a total number of 68 patients, with 34 clinically and histopathologically confirmed leprosy patients and 34 age- and sex-matched healthy controls attending Maharishi Markandeshwar Institute of Medical Sciences and Research, a tertiary care centre in North India. Serum IL-17 and IL-22 levels were measured using an enzyme-linked immunosorbent assay. Patients were classified according to the Ridley-Jopling spectrum, and reactional states were analysed separately.

Results: Mean serum IL-17 and IL-22 levels were significantly higher in leprosy patients than in controls (p = 0.001 and p < 0.001, respectively). Both cytokines varied significantly across the Ridley-Jopling spectrum (p < 0.001). The highest IL-17 levels were observed in erythema nodosum leprosum and reversal reaction (n = 10 (29.4%)), whereas IL-22 levels were markedly elevated in borderline and reactional forms. IL-22 levels were significantly higher in slit skin smear-negative patients (n = 14 (41.2%)), while IL-17 did not show a significant association with smear status.

Conclusion: Elevated serum IL-17 and IL-22 levels, particularly in borderline and reactional forms of leprosy, support a contributory role of the Th17/Th22 axis in disease immunopathogenesis and highlight their potential utility as biomarkers of inflammatory activity in lepra reactions.

## Introduction

Leprosy is a chronic infectious granulomatous disease caused by *Mycobacterium leprae*, an obligate intracellular bacillus that continues to pose a significant public health challenge in several endemic regions, including India, South America, Central Africa, and South East Asia [1]. Transmission occurs predominantly through respiratory droplets from untreated multibacillary patients, with humans serving as the principal reservoir and the armadillo being the only established non-human host [2]. According to recent World Health Organization (WHO) data, the global number of newly detected leprosy cases decreased further from 182,815 in 2023 to 172,717 in 2024, continuing the long-term downward trend in annual incidence. However, significant transmission persists in endemic regions, and a substantial proportion of newly detected cases still present with grade 2 disability, underscoring ongoing challenges in early detection and control. India continues to contribute the largest share of the global leprosy burden. In 2024-2025, a total of 100,957 new cases were detected, corresponding to an Annual New Case Detection Rate (ANCDR) of 7.00 per 100,000 population. As of March 31, 2025, 82,297 cases remained under treatment, with a prevalence rate of 0.57 per 10,000 population. Of the newly detected cases, 63.03% were multibacillary, 40.07% were females, and 4.68% were children, while the grade 2 disability rate among new cases was 1.88% [3].

Leprosy has a spectrum of varied clinical presentations. It has been classified by Ridley and Jopling on the basis of histological and immunological features into five types [4]. The frequent presentation is an area with loss of sensation on the skin or a visible hypopigmented or hyperpigmented anesthetic or hypoesthetic skin lesion. Tuberculoid leprosy presents with few, well-defined hypopigmented anesthetic lesions due to strong cell-mediated immunity. Mid-borderline leprosy is an unstable form with dimorphic erythematous plaques. Lepromatous leprosy shows multiple, symmetrical papules and nodules with diffuse infiltration, severe nerve involvement, and deformity. Nodular and diffuse forms of lepromatous disease have been observed [5]. Nerve involvement in leprosy ranges from intradermal nerve damage to involvement of major peripheral nerve trunks, with enlargement of superficial nerves. Sensory loss may involve touch, pain, or temperature, and neuropathy can be silent or present with weakness, atrophy, and deformities, with glove-and-stocking sensory loss seen in lepromatous leprosy [6]. Polar leprosy is stable, whereas borderline forms commonly develop reactions: type 1 (reversal) is a delayed hypersensitivity reaction, and type 2 (erythema nodosum leprosum) is an immune complex-mediated acute inflammatory reaction [7].

The clinical spectrum of leprosy is determined primarily by the host immune response to Mycobacterium leprae rather than by the organism itself. Strong cell-mediated immunity results in the tuberculoid form, characterized by few bacilli and lymphocyte-rich granulomas, whereas defective cellular immunity leads to lepromatous leprosy with heavy bacillary load and macrophage-dominant infiltrates [8]. Innate immunity via complement receptors and Toll-like receptors initiates the response, while adaptive immunity, particularly Th1 and Th2 polarization, governs disease expression and reactions [9]. Recent evidence highlights the role of Th17 and Th22 pathways, with cytokines such as interleukin (IL)-17 and IL-22 contributing to inflammation, tissue response, and disease progression, especially during reactions [10]. Despite extensive data on Th1/Th2 responses, the role of Th17-related cytokines remains underexplored. Targeting IL-17 and IL-22 can be a future helpful approach to limit this endemic disabling disease. Hence, the present study was conducted to assess the role of IL-17 and IL-22 in leprosy.

## Materials and methods

Study design

This is a cross-sectional study.

Study area

This study was conducted on 68 patients (34 cases of leprosy and 34 age- and sex-matched controls), after assessing their eligibility according to the selection criteria, attending the dermatology OPD at Maharishi Markandeshwar Institute of Medical Sciences and Research, Mullana, Ambala, India. The study was conducted over an 18-month period from January 2019 to July 2020.

Inclusion Criteria

This study included patients of either sex and any age diagnosed with leprosy on clinical grounds and confirmed histopathologically.

Exclusion Criteria

Patients receiving systemic corticosteroids, thalidomide, or immunomodulatory therapy within the preceding four weeks were excluded to minimize treatment-related cytokine modulation. Individuals with known autoimmune disease, chronic infections, diabetes mellitus with active inflammation, or other systemic inflammatory conditions were also excluded. 

Controls

The control group comprised attendants of patients attending the hospital, who were age- and sex-matched with cases prior to enrollment.

Strategy

Written informed consent was obtained from all cases and controls willing to participate in the study. Clinical cases of leprosy were biopsied, and the diagnosis was confirmed both clinically and histopathologically. After fulfilling the inclusion criteria, a detailed medical history and thorough physical examination were conducted. A predesigned and prestructured proforma was completed for each participant, which included details of presenting complaints, duration of illness, prior medications, family history, personal history, associated illnesses, and the areas involved. Based on the diagnosis, the disease was classified according to the Ridley-Jopling classification into the conventional five groups (Group 1: TT, Group 2: BT, Group 3: BB, Group 4: BL, Group 5: LL, Group 6: RR, Group 7: ENL). However, cases presenting with lepra reactions were analyzed separately.

Analysis of samples/measurement of interleukins

Serum IL-17 and IL-22 levels were measured using commercially available sandwich ELISA kits from Diaclone, France (Medix Biochemica Group) for IL-17 and IL-22. The analytical sensitivity of the assays was 2 pg/mL for IL-17 and 5 pg/mL for IL-22, with a measurable range of 7.8-500 pg/mL for both cytokines, as specified by the manufacturer. The intra-assay and inter-assay coefficients of variation were <8% and <10%, respectively.

Venous blood samples (5 mL) were collected under aseptic precautions, centrifuged at 3000 rpm for 10 minutes, and serum was aliquoted into sterile cryovials and stored at −80 °C until analysis. Samples were thawed only once to avoid cytokine degradation, and all measurements were performed in duplicate according to the manufacturer’s protocol. Standard calibration curves were generated for each plate, and optical density was read at 450 nm with 620 nm reference using a microplate ELISA reader. Internal quality controls supplied with the kit were run with each batch.

Analysis of data

IBM SPSS Statistics for Windows, version 28.0 (released 2021, IBM Corp., Armonk, NY) was used to analyze the data. Normality of continuous variables was assessed using the Shapiro-Wilk test and inspection of Q-Q plots. Comparison of cytokine levels between cases and controls was performed using the independent samples t-test for normally distributed data and the Mann-Whitney U test where normality was violated. Comparison across multiple diagnostic subgroups was performed using one-way ANOVA with Tukey post hoc correction for normally distributed variables; otherwise, the Kruskal-Wallis test with Dunn-Bonferroni adjustment was applied. The association between clinical type and slit-skin smear was assessed using the Chi-square test. A two-tailed p-value <0.05 was considered statistically significant.

Ethical considerations

Ethical approval was obtained from the Institutional Ethics Committee of Maharishi Markandeshwar Institute of Medical Science and Research prior to the commencement of the study (approval no. IEC-259). Patients were enrolled after receiving a proper explanation about this study, and informed consent was obtained, reassuring confidentiality of the information shared.

## Results

In 34 cases of leprosy, the age ranged from 22 to 75 years, with a mean age of 40.35 ± 14.24 years. Most of the cases, that is 12 (35.3%), were between the ages of 41 and 50 years, while the least number of cases, that is 5 (14.7%), were more than 50 years of age. The control group was matched with the cases by age. The mean age of the controls was 39.94 ± 13.37 years. Among the cases, 19 (55.9%) were males, and 15 (44.1%) were females. The control group was sex matched. The male-to-female ratio was 1.26:1.

Among the cases, housewives (11; 32.3%) formed the largest occupational group, followed by unskilled workers (9; 26.5%) and semi-skilled/skilled workers (7; 20.5%); by contrast, most controls (15; 44.1%) were semi-skilled or skilled workers. Most patients (13; 38.2%) presented within ≤3 months of disease onset, while four patients (11.8%) had disease duration exceeding 12 months. 

Leprosy was classified according to the Ridley-Jopling classification, with cases presenting with lepra reactions analyzed separately. Among the 34 cases, the most common subtype was lepromatous leprosy, seen in 10 patients (29.4%), while the least common was mid-borderline leprosy, seen in two patients (5.9%). Borderline lepromatous leprosy and tuberculoid leprosy were observed in three patients (8.8%) each, and borderline tuberculoid leprosy was noted in six patients (17.6%). Type 1 and type 2 lepra reactions were present in five patients (14.7%) each. Out of the 34 leprosy cases, 20 patients (58.8%) were slit skin smear positive, while 14 patients (41.2%) were slit skin smear negative. Among the study population, five patients (14.7%) had a type 1 reaction, and five patients (14.7%) had a type 2 reaction (Table 1). 

**Table 1:** Distribution of the patients according to diagnosis.

Diagnosis	Frequency	Percentage(%)
Tuberculoid leprosy	3	8.8
Borderline tuberculoid	6	17.6
Mid borderline	2	5.9
Borderline lepromatous	3	8.8
Lepromatous leprosy	10	29.4
Reversal reaction	5	14.7
Erythema nodosum leprosum	5	14.7

A statistically significant association was observed between the clinical type of leprosy and slit skin smear status (χ² = 27.495, df = 6, p < 0.001). All patients with lepromatous leprosy (n = 10), borderline lepromatous leprosy (n = 3), and erythema nodosum leprosum (n = 5) were slit skin smear positive (100%), whereas tuberculoid leprosy (n = 3), mid-borderline leprosy (n = 2), and reversal reaction (n = 5) showed 100% slit skin smear negativity. Among borderline tuberculoid leprosy, two patients (33.3%) were slit skin smear positive, while four patients (66.7%) were negative (Table 2). 

**Table 2:** Diagnosis of the patients according to slit smear.

Type of leprosy	N	Positive (n = 20) F (%)	Negative (n = 14) F(%)	X^2^	df	p-value
Tuberculoid leprosy	3	0 (0%)	3 (100%)	27.495^#^	6	0.000^ S^
Borderline tuberculoid leprosy	6	2(33.3%)	4 (66.7%)	27.495^#^	6	0.000^ S^
Mid borderline leprosy	2	0(0%)	2 (100%)	27.495^#^	6	0.000^ S^
Borderline lepromatous leprosy	3	3(100%)	0 (0%)	27.495^#^	6	0.000^ S^
Lepromatous leprosy	10	10(100%)	0 (0%)	27.495^#^	6	0.000^ S^
Reversal reaction	5	0(0%)	5 (100%)	27.495^#^	6	0.000^ S^
Erythema nodosum leprosum	5	5(100%)	0 (0%)	27.495^#^	6	0.000^ S^

The mean serum IL-17 level in leprosy patients (192.97 pg/mL) was higher than that observed in controls (78.18 pg/mL). The mean difference between the two groups was 114.79 pg/mL, which was statistically significant (p = 0.001). Similarly, the mean serum IL-22 level was significantly higher in leprosy cases (244.88 pg/mL) compared to controls (62.35 pg/mL), with a mean difference of 182.53 pg/mL (p < 0.001) (Table 3). 

**Table 3:** Mean difference in IL-17 (pg/ml) and IL-22 (pg/ml) between the cases and controls.

Parameter	IL-17 (pg/mL)	IL-22 (pg/mL)
Mean in cases	192.97	244.88
Mean in controls	78.18	62.35
Mean difference	114.794	182.529
p-value	0.001	0.000
Significance	Significant	Significant

The mean IL-17 level among the slit skin smear-positive patients was 209.95 ± 254.34 pg/mL, while it was 168.71 ± 54.76 pg/mL among the slit skin smear-negative patients. This difference was not statistically significant. By contrast, the mean IL-22 level was 144.75 ± 113.11 pg/mL in the slit skin smear-positive patients and 387.90 ± 177.17 pg/mL in the slit skin smear-negative patients. This difference was found to be statistically significant (Table 4). 

**Table 4:** Mean difference in IL-17 (pg/ml) and IL-22 (pg/ml) according to the slit skin smear.

Parameter	IL-17 (pg/mL)	IL-22 (pg/mL)
Mean in slit smear-positive patients	209.95 ± 254.34	144.75 ± 113.11
Mean in slit smear-negative patients	168.71 ± 54.76	387.90 ± 177.17
p-value	>0.05	<0.05
Significance	Non-significant	Significant

Mean serum IL-17 levels varied significantly across the Ridley-Jopling spectrum (p < 0.001), with the lowest levels observed in lepromatous leprosy (42.70 ± 10.60 pg/mL). Progressive increases were noted from borderline lepromatous (88.33 ± 9.60 pg/mL) and mid-borderline leprosy (115.50 ± 7.77 pg/mL) to tuberculoid (130.00 ± 36.66 pg/mL) and borderline tuberculoid leprosy (157.50 ± 36.14 pg/mL). Markedly elevated levels were seen in the reversal reaction (227.60 ± 28.07 pg/mL), with the highest levels in erythema nodosum leprosum (633.00 ± 32.53 pg/mL). Similarly, IL-22 levels showed significant variation across clinical subtypes (p < 0.001). Lower levels were observed in lepromatous (82.10 ± 12.50 pg/mL) and tuberculoid leprosy (82.00 ± 14.42 pg/mL), with higher levels in borderline lepromatous leprosy (99.67 ± 9.71 pg/mL). Marked elevations were noted in borderline tuberculoid (451.67 ± 71.91 pg/mL), mid-borderline leprosy (459.00 ± 26.87 pg/mL), and reversal reaction (490.80 ± 71.17 pg/mL), while erythema nodosum leprosum showed moderately elevated IL-22 levels (175.60 ± 41.44 pg/mL) (Table 5). 

**Table 5:** Mean difference in IL-17 (pg/ml) and IL-22 (pg/ml) according to diagnosis.

Diagnosis	IL-17 (pg/mL) Mean ± SD	IL-22 (pg/mL) Mean ± SD
Tuberculoid leprosy	130.00 ± 36.66	82.00 ± 14.42
Borderline tuberculoid leprosy	157.50 ± 36.14	451.67 ± 71.91
Mid-borderline leprosy	115.50 ± 7.77	459.00 ± 26.87
Borderline lepromatous leprosy	88.33 ± 9.60	99.67 ± 9.71
Lepromatous leprosy	42.70 ± 10.60	82.10 ± 12.50
Reversal reaction	227.60 ± 28.07	490.80 ± 71.17
Erythema nodosum leprosum	633.00 ± 32.53	175.60 ± 41.44
F value	318.465	87.419
p-value	0.000	0.000
Significance	Significant	Significant

With respect to disease duration, the mean IL-17 level was highest in patients with a disease duration of <3 months (285.69 ± 239.65 pg/mL) and lowest in those with durations between six and nine months (87.60 ± 49.31 pg/mL). However, the difference across duration groups was statistically non-significant (p = 0.114). Similarly, for IL-22, the highest mean level was observed in patients with a disease duration of nine to 12 months (389.40 ± 178.01 pg/mL), while the lowest mean was noted in those with a duration between six and nine months (88.00 ± 14.63 pg/mL). The variation across disease duration groups was not statistically significant (p = 0.104) (Table 6). 

**Table 6:** Mean difference in IL-17 (pg/ml) and IL-22 (pg/ml) according to the duration of disease.

Duration of disease (months)	IL-17 (pg/mL) Mean ± SD	IL-22 (pg/mL) Mean ± SD
≤ 3	285.69 ± 239.65	269.77 ± 188.33
3–6	95.00 ± 63.12	249.57 ± 204.90
6–9	87.60 ± 49.31	88.00 ± 14.63
9–12	128.80 ± 69.65	389.40 ± 178.01
>12	275.00 ± 293.18	171.25 ± 158.85
F value	2.044	2.119
p-value	0.114	0.104
Significance	Non-significant	Non-significant

Post-hoc comparison of IL-17 levels across different disease-duration groups showed a statistically significant difference only between patients with disease duration ≤3 months and three to six months (mean difference 190.69 ± 87.06 pg/mL; p = 0.037). All other pairwise comparisons for IL-17 were not statistically significant. For IL-22, a statistically significant difference was observed only between patients with disease duration of six to nine months and nine to 12 months (mean difference −301.40 ± 110.22 pg/mL; p = 0.011). The remaining intergroup comparisons did not show statistically significant differences (Table 7). 

**Table 7:** Post-hoc comparison for IL-17 (pg/ml) and IL-22 (pg/ml) according to the duration of disease.

Duration comparison (months)	IL-17 mean difference ± SE (pg/mL)	IL-17 p-value	IL-22 mean difference ± SE (pg/mL)	IL-22 p-value
≤ 3 vs. 3–6	190.69 ± 87.06	0.037 ^(S)^	20.20 ± 81.70	0.806
≤ 3 vs. 6–9	198.09 ± 97.73	0.052	181.77 ± 91.71	0.057
≤ 3 vs. 9–12	156.89 ± 97.73	0.119	−119.63 ± 91.71	0.202
≤ 3 vs. >12	10.69 ± 106.18	0.920	98.52 ± 99.65	0.331
3–6 vs. 6–9	7.40 ± 108.74	0.946	161.57 ± 102.05	0.124
3–6 vs. 9–12	−33.80 ± 108.74	0.758	−139.83 ± 102.05	0.181
3–6 vs. >12	−180.00 ± 116.40	0.133	78.32 ± 109.23	0.479
6–9 vs. 9–12	−41.20 ± 117.45	0.728	−301.40 ± 110.22	0.011 ^(S)^
6–9 vs. >12	−187.40 ± 124.58	0.143	−83.25 ± 116.91	0.482
9–12 vs. >12	−146.20 ± 124.58	0.250 (NS)	218.15 ± 116.91	0.072

Post-hoc analysis demonstrated significant intergroup differences in both the IL-17 and IL-22 levels across the Ridley-Jopling spectrum and lepra reactions. For IL-17, tuberculoid and borderline tuberculoid forms showed significantly higher levels compared to lepromatous leprosy, while reversal reaction and erythema nodosum leprosum (ENL) exhibited markedly elevated levels when compared with all non-reactional forms (p < 0.001 for most comparisons). ENL consistently showed significantly higher IL-17 levels than all other diagnostic categories. For IL-22, significant differences were observed mainly between borderline and reactional states. Borderline tuberculoid and mid-borderline leprosy showed significantly higher IL-22 levels compared to tuberculoid and lepromatous leprosy (p < 0.001). Reversal reaction demonstrated significantly elevated IL-22 levels compared to tuberculoid, borderline lepromatous, and lepromatous leprosy, while ENL also differed significantly from several non-reactional forms. Comparisons between closely related non-reactional groups were largely not statistically significant (Table 8). 

**Table 8:** Post-hoc comparison for IL-17 (pg/ml) and IL-22 (pg/ml) between various diagnoses.

Diagnostic comparison	IL-17 mean difference ± SE (pg/mL)	IL-17 p-value	IL-22 mean difference ± SE (pg/mL)	IL-22 p-value
Tuberculoid leprosy vs. borderline tuberculoid leprosy	−27.50 ± 18.19	0.142	−369.67 ± 32.12	0.000 ^(S)^
Tuberculoid leprosy vs. mid-borderline tuberculoid leprosy	14.50 ± 23.48	0.542	−377.00 ± 41.47	0.000 ^(S)^
Tuberculoid leprosy vs. borderline lepromatous leprosy	41.67 ± 21.00	0.058	−17.67 ± 37.09	0.638
Tuberculoid leprosy vs. lepromatous leprosy	87.30 ± 16.93	0.000 ^(S)^	−0.10 ± 29.91	0.997
Tuberculoid leprosy vs. reversal reaction	−97.60 ± 18.79	0.000 ^(S)^	−408.80 ± 33.18	0.000 ^(S)^
Tuberculoid leprosy vs. erythema nodosum leprosum	−503.00 ± 18.79	0.000 ^(S)^	−93.60 ± 33.18	0.009 ^(S)^
Borderline tuberculoid leprosy vs. mid-borderline tuberculoid leprosy	42.00 ± 21.00	0.056	−7.33 ± 37.09	0.845
Borderline tuberculoid leprosy vs. borderline lepromatous leprosy	69.17 ± 18.19	0.001 ^(S)^	352.00 ± 32.12	0.000^ (S)^
Borderline tuberculoid leprosy vs. lepromatous leprosy	114.80 ± 13.28	0.000 ^(S)^	369.57 ± 23.46	0.000 ^(S)^
Borderline tuberculoid leprosy vs. reversal reaction	−70.10 ± 15.58	0.000 ^(S)^	−39.13 ± 27.51	0.166
Borderline tuberculoid leprosy vs. erythema nodosum leprosum	−475.50 ± 15.58	0.000 ^(S)^	276.07 ± 27.51	0.000 ^(S)^
Mid-borderline tuberculoid leprosy vs. borderline lepromatous leprosy	27.17 ± 23.48	0.257	359.33 ± 41.47	0.000 ^(S)^
Mid-borderline tuberculoid leprosy vs. lepromatous leprosy	72.80 ± 19.92	0.001 ^(S)^	376.90 ± 35.19	0.000 ^(S)^
Mid-borderline tuberculoid leprosy vs. reversal reaction	−112.10 ± 21.52	0.000 ^(S)^	−31.80 ± 38.01	0.410
Mid-borderline tuberculoid leprosy vs. erythema nodosum leprosum	−517.50 ± 21.52	0.000 ^(S)^	283.40 ± 38.01	0.000 ^(S)^
Borderline lepromatous leprosy vs. lepromatous leprosy	45.63 ± 16.93	0.012 ^(S)^	17.57 ± 29.91	0.562
Borderline lepromatous leprosy vs. reversal reaction	−139.27 ± 18.79	0.000 ^(S)^	−391.13 ± 33.18	0.000 ^(S)^
Borderline lepromatous leprosy vs. erythema nodosum leprosum	−544.67 ± 18.79	0.000 ^(S)^	−75.93 ± 33.18	0.030^ (S)^
Lepromatous leprosy vs. reversal reaction	−184.90 ± 14.09	0.000 ^(S)^	−17.57 ± 29.91	0.562
Lepromatous leprosy vs. erythema nodosum leprosum	−590.30 ± 14.09	0.000 ^(S)^	−408.70 ± 24.88	0.000 ^(S)^
Reversal reaction vs. erythema nodosum leprosum	−405.40 ± 16.27	0.000 ^(S)^	315.20 ± 28.73	0.000 ^(S)^

## Discussion

Leprosy represents a unique immunological model in which the clinical spectrum and disease activity are dictated primarily by the host immune response rather than the bacillary load. This immunological heterogeneity underlies the Ridley-Jopling classification [4], ranging from tuberculoid leprosy with strong cell-mediated immunity to lepromatous leprosy characterized by immune anergy and high bacillary burden. Reactional states and borderline forms are characterized by immunological instability, resulting in sudden shifts in cytokine balance and exaggerated inflammatory responses. In recent years, increasing attention has been directed toward the role of the Th17/Th22 axis in leprosy, particularly in reactional states. The findings of the present study, demonstrating elevated serum levels of IL-17 and IL-22 in reactional and unstable forms of leprosy, are in concordance with and further extend observations from earlier immunological studies.

Initial insights into the role of IL-17 in leprosy were provided by studies demonstrating enhanced Th17 responses in borderline and reactional forms of the disease. Saini et al. [11] reported increased IL-17 expression in skin lesions of borderline leprosy compared to stable polar forms, suggesting that IL-17 contributes to immunological instability and heightened inflammation in these patients. These findings support the concept that IL-17 plays a critical role in amplifying inflammatory cascades during reactional episodes.

The pro-inflammatory nature of IL-17 is well established, with its ability to recruit neutrophils, activate macrophages, and induce the production of downstream cytokines such as TNF-α, IL-6, and IL-1β. In the context of leprosy, increased IL-17 may act synergistically with Th1 cytokines, particularly interferon-γ, to intensify tissue inflammation. De Sousa et al. [12] observed a mixed Th1-Th17 cytokine profile in borderline leprosy, highlighting the coexistence of protective and pathogenic immune responses in unstable disease. This dual immune activation may explain the paradox of effective bacillary control alongside progressive tissue and nerve damage seen in reactional states.

IL-22, a cytokine closely linked to Th17 and Th22 cells, has also emerged as an important mediator in leprosy immunopathogenesis. Although IL-22 is traditionally regarded as a tissue-protective cytokine involved in epithelial regeneration and antimicrobial defense, its dysregulated expression has been implicated in chronic inflammatory dermatoses such as psoriasis and atopic dermatitis. In leprosy, studies by Costa et al. [13] demonstrated increased IL-22 expression in reactional lesions, particularly erythema nodosum leprosum, suggesting its involvement in epidermal hyperplasia, dermal edema, and inflammatory infiltration. The elevated IL-22 levels observed in the present study further support its role in the cutaneous manifestations of lepra reactions.

Experimental data suggest that IL-22 acts primarily on keratinocytes and stromal cells, inducing the production of antimicrobial peptides and pro-inflammatory mediators. In reactional leprosy, this may contribute to exaggerated skin inflammation and clinical worsening despite effective antimicrobial therapy. Furthermore, the combined action of IL-17 and IL-22 may perpetuate a self-sustaining inflammatory loop, leading to prolonged reactional episodes and increased risk of nerve involvement. This hypothesis is supported by findings from Sadhu S et al. [14] who demonstrated co-expression of IL-17 and IL-22 in reactional lesions, indicating activation of a coordinated Th17/Th22 immune response.

Serum-based analyses by Abdallah M et al. [15] revealed significantly higher IL-17 levels in patients experiencing lepra reactions compared to non-reactional patients, suggesting that circulating cytokine levels may reflect underlying immunological activity. Several studies [11-15] have also highlighted the potential utility of these cytokines as biomarkers of disease activity. Our findings align with these observations and indicate that measurement of IL-17 and IL-22 may aid in identifying patients at risk of developing reactions, particularly in borderline forms of the disease.

The clinical implications of these findings are significant. Lepra reactions remain the primary cause of nerve damage and disability in leprosy, and their unpredictable nature poses a major therapeutic challenge. Current management relies heavily on systemic corticosteroids, which are associated with substantial adverse effects when used long term. The growing evidence implicating IL-17 and IL-22 in reactional pathology raises the possibility of targeted immunomodulatory therapy. Biological agents targeting the IL-17 pathway have shown efficacy in other inflammatory dermatoses and may, in the future, offer a more selective approach to controlling inflammation in lepra reactions. Key agents include Secukinumab, Ixekizumab (target IL-17A), Brodalumab (blocks receptor), and Bimekizumab (targets A and F isoforms). Although such strategies remain exploratory, they underscore the importance of understanding cytokine networks in disease pathogenesis.

In summary, the present study adds to the growing body of evidence supporting a central role for the Th17/Th22 axis in the immunopathogenesis of reactional and unstable forms of leprosy. Elevated IL-17 and IL-22 levels reflect heightened inflammatory activity and immunological imbalance, contributing to tissue and nerve damage. Integrating our findings with previous studies, it is evident that these cytokines are not only markers of disease activity but may also represent potential therapeutic targets. Further longitudinal and mechanistic studies are warranted to clarify their predictive value and role in guiding future treatment strategies. This study has certain limitations. The relatively small sample size and the single-centre, cross-sectional design restrict the generalisability of the findings and do not permit evaluation of temporal changes or treatment response. Furthermore, circulating serum cytokine levels may not fully represent local cutaneous immune activity, which could influence the interpretation of immunological associations. Residual confounding due to subclinical infections or unrecognised inflammatory conditions cannot be entirely excluded. In addition, the study was primarily powered to address the main objectives and was not specifically designed for multiple subgroup or post-hoc analyses; consequently, some of these comparisons may be underpowered with a potential risk of type II error.

## Conclusions

This study demonstrates significantly elevated serum levels of IL-17 and IL-22 in patients with leprosy compared to healthy controls, with variation across the Ridley-Jopling spectrum. Higher cytokine levels observed in borderline forms and lepra reactions indicate an association between the Th17/Th22 axis and inflammatory disease activity, supporting their involvement in immunological instability in leprosy. However, these findings reflect serum-based associations and should not be interpreted as evidence of causality or validated clinical biomarkers. The potential utility of IL-17 and IL-22 as indicators of reactional states or as therapeutic targets remains exploratory and hypothesis-generating. Further large-scale, longitudinal, and tissue-correlative studies are required to confirm these observations, determine their predictive value, and evaluate any future clinical applicability.
